# Hospital autopsy: Endangered or extinct?

**DOI:** 10.1136/jclinpath-2014-202700

**Published:** 2015-06-15

**Authors:** Angus Turnbull, Michael Osborn, Nick Nicholas

**Affiliations:** 1 Imperial College School of Medicine, London, UK; 2 Department of Cellular Pathology, Imperial College Healthcare NHS Trust, London, UK; 3 Hillingdon Hospital Foundation Trust, Uxbridge, London, UK

**Keywords:** AUTOPSY PATHOLOGY, POSTMORTEM, MEDICAL LAW, MEDICAL STATISTICS, MEDICAL EDUCATION

## Abstract

**Aim:**

To determine the hospital autopsy rate for the UK in 2013.

**Methods:**

A study of data from a ‘Freedom of Information’ request to all (n=186) acute NHS Trusts within England (n=160), NHS Boards in Scotland (n=14) and Wales (n=7) and Social Care Trusts in Northern Ireland (n=5). Hospital autopsy rates were calculated from the number of hospital autopsies performed in 2013 as a percentage of total inpatient deaths in the Trust that year.

**Results:**

The UK response rate was 99% (n=184), yielding a mean autopsy rate of 0.69%. The mean rates were 0.51% (England), 2.13% (Scotland), 0.65% (Wales) and 0.46% (Northern Ireland). 23% (n=38) of all included respondents had a rate of 0% and 86% (n=143) a rate less than 1%.

**Conclusions:**

The decline in hospital autopsy has continued relentlessly and, for better or for worse, the practice is on the verge of extinction in the UK. The study highlights to health professionals and policy makers the magnitude of this decline. Further research should investigate the impact of this on patient safety, clinical audit, public health and medical education.

## Introduction

Autopsy from the Greek ‘autos’ and ‘opsomeri’ means ‘to see for oneself’.^1^ ^2^ Its history stems from mummification and human dissection in 3000 BC, through ancient Greece where Hirophilus discovered the duodenum by live human dissection to Rokitansky (1804–1878), regarded as the father of the modern autopsy and who performed or supervised over 100 000 examinations.^1^

Autopsies in the UK comprise medicolegal (those required by HM coroner or in Scotland the procurator fiscal) and hospital consent (clinical) autopsies. Many doctors believe that autopsy is outdated while some argue that autopsies should remain an integral part of medicine, education, clinical audit and research.^1^

In 2013, 45% of registered deaths in England and Wales were reported to the coroner. Of these, 41% underwent coronial autopsy, accounting for approximately 20% of all deaths and over 94 000 autopsies.^3^

**Figure 1:**
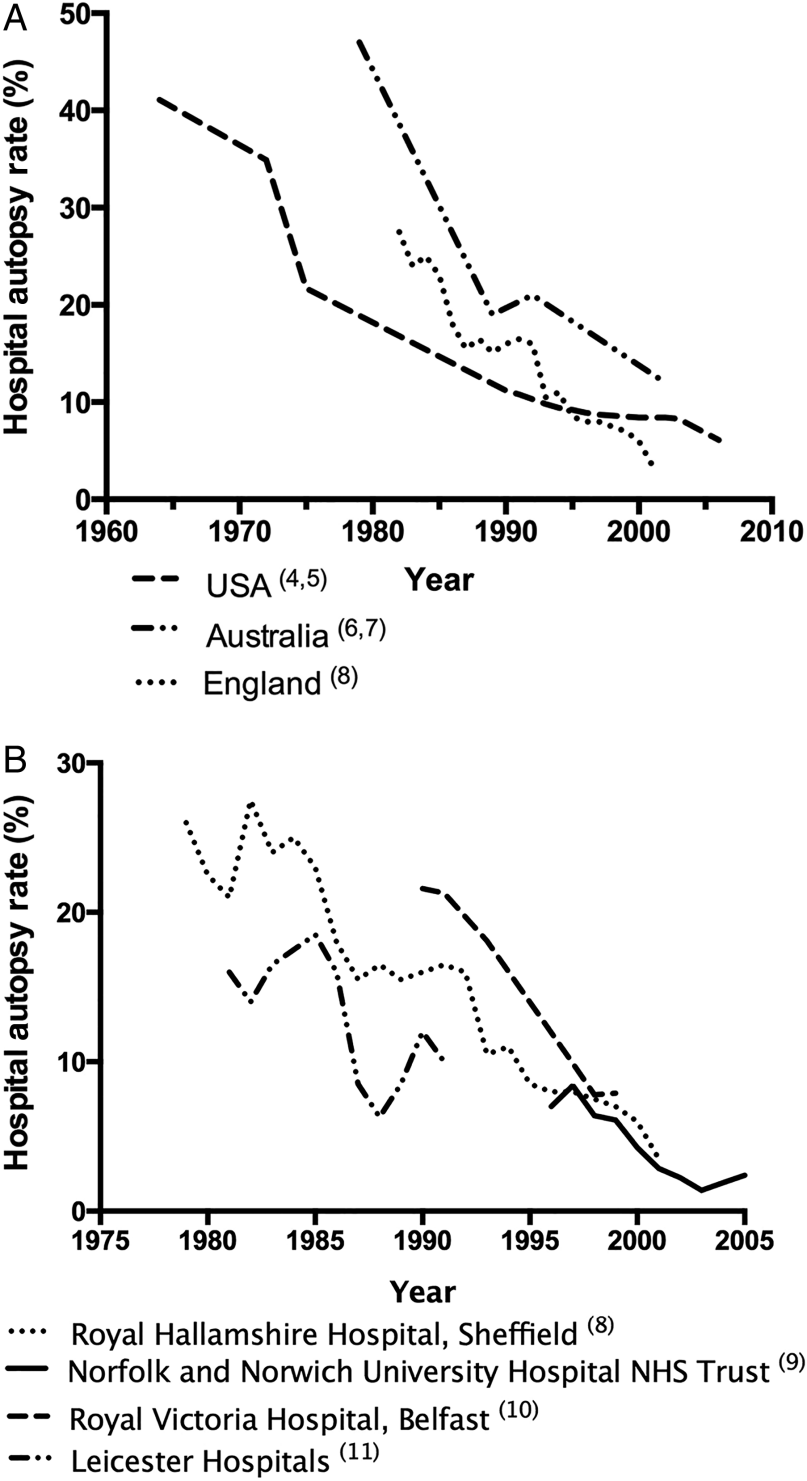
Decline in hospital autopsy rates over recent decades. (A) Autopsy rates from three first world countries, data collated from multiple studies. (B) Autopsy rates from four different hospitals/NHS Trusts, data collated from multiple studies.

Hospital autopsy rates have been falling in the UK and worldwide for over half a century^4–15^ (figure 1A, B) and account for a small minority of all autopsies in the UK.^1^ ^3^ Recent studies suggest autopsy rates of less than 10% for teaching hospitals and less than 5% elsewhere.^1^ ^16^

The decline in hospital autopsy rates is well known, yet poorly researched and quantified. The majority of medical professionals and politicians in the UK are likely to be unaware of this conspicuous decline. Consequently, little has been done to address the falling rates and the implications of this are not yet fully understood, nor are the consequences.

A PubMed literature search yielded no research detailing a UK-wide autopsy rate within the past 20 years (search terms “hospital autopsy [title]”, “clinical autopsy [title]”, “autopsy rate [title]”). Given this and documented inter-hospital variation (figure 1B), we aimed to determine the current UK autopsy rate.

The structure of healthcare delivery varies throughout the UK. In England, the provision of acute services (emergency, inpatient and outpatient care) is provided by 186 organisations known as Acute National Health Service (NHS) Trusts—each of which may provide care from multiple hospital sites. In Scotland and Wales, the countries are divided into a number of defined geographical areas (Boards), each of which may contain several sites of healthcare delivery. In Northern Ireland, these geographical areas are known as Health and Social Care Trusts.

## Method

Acute NHS Trusts within England (n=160), Boards within Wales (n=7) and Scotland (n=14) and Social Care Trusts within Northern Ireland (n=5) were contacted via ‘Freedom of Information’ requests. The level of response therefore is for the Trust/Board, not individual hospitals. If no reply was received within 4 months, reminders were sent.

The hospital autopsy rate was calculated as the number of autopsies performed on patients who died in the year 2013 as a percentage of total deaths which occurred in the hospital in that calendar year.

Studies indicate significantly higher autopsy rates in stillbirths, neonates and young children.^17^ ^18^ Therefore, data were excluded if they fell within the following categories:

1. Children's Hospital NHS Trusts
2. Stillbirth, neonatal, perinatal and paediatric death
3. Trusts with no recorded deaths
4. Incomplete responses

Statistical analysis was performed using two-tailed χ^2^ tests (Prism 6 Software) between each country. The categories used were number of deaths that underwent autopsy and number of deaths not followed by autopsy. Bonferroni correction was used to compensate for the six pairwise comparisons, resulting in 99.25 CIs (p<0.008). Statistical outliers were determined with a ROUT test using a false-positive rate (Q) of 1%.

## Results

A 99% (n=184) response rate was achieved for the UK; constituent country response rates were 99% (England), 100% (Scotland), 100% (Wales) and 100% (Northern Ireland). A total of 17 Trusts were removed, according to the exclusion criteria. Eight Trusts were concerned about patient identification because the number of autopsies was small and so provided a ‘fewer than’ figure. In these cases, a maximum possible rate was calculated.

**Figure 2:**
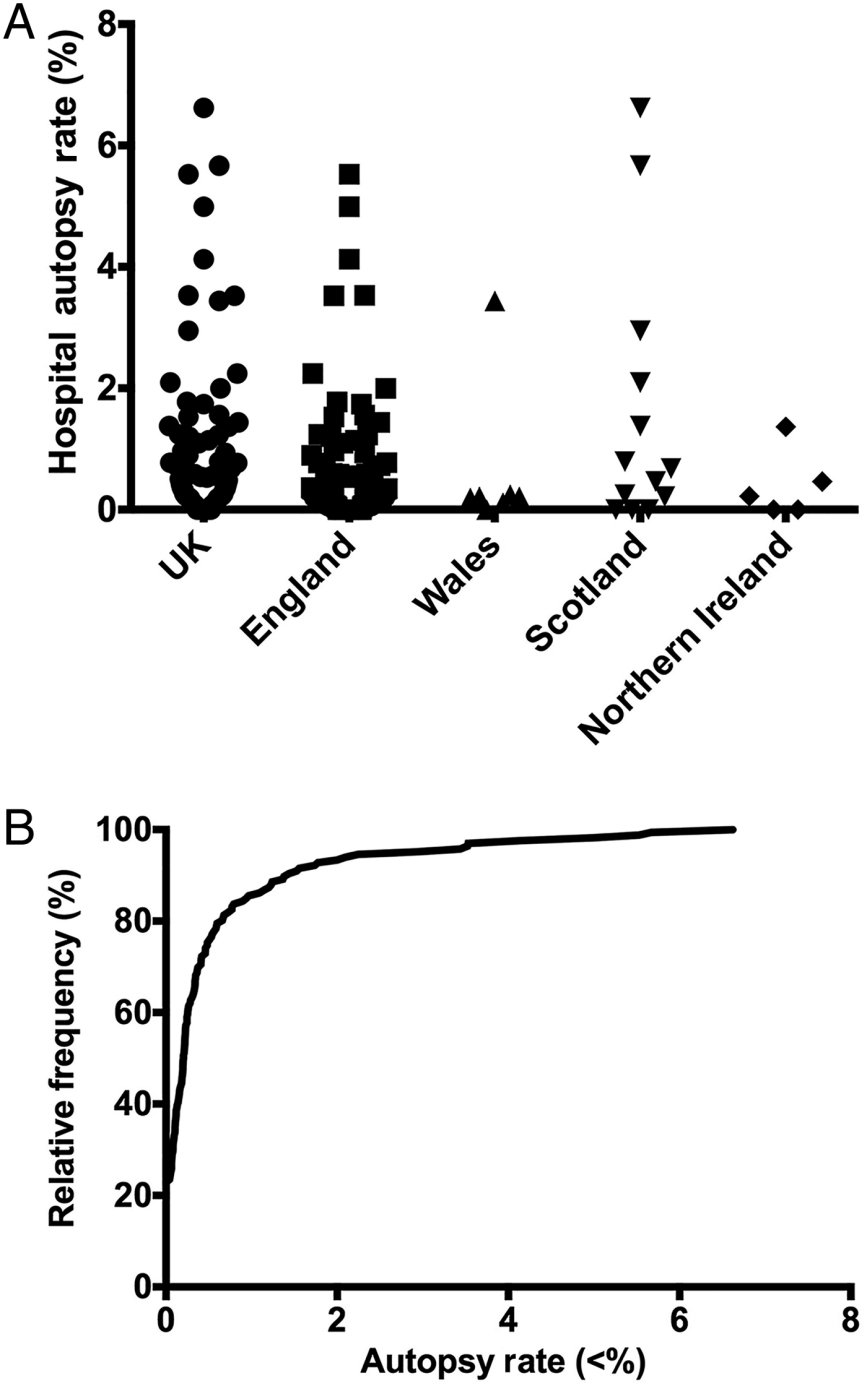
The results from Freedom of Information request for UK and constituent countries. (A) Individual points representing each sample Trust/Board, non-parametric data, no statistical difference between countries. (B) Cumulative frequency histogram of autopsy rates for NHS Trusts/Boards in the UK.

Mean hospital autopsy rates were calculated as the total number of autopsies expressed as a percentage of the total number of deaths. The UK mean autopsy rate was 0.69% and varied considerably between countries. The highest mean autopsy rates were in Scotland (2.1%), followed by Wales (0.65%), England (0.51%) and Northern Ireland (0.46%). The study confirms that hospital autopsy rates are significantly lower than the most recent literature suggests and that there is evident inter-country variation (figure 2A, table 1) and intra-country variation (figure 2A).

**Table 1:** Summary of response rate and mean autopsy rate for the UK and each constituent country

Region	n	Response rate (%)	Excluded Trusts	Total deaths	Total autopsies	Mean autopsy rate (%)
UK	186	99	17	252 185	1734	0.69
England	160	99	16	202 518	1027	0.51
Scotland	14	100	1	26 909	572	2.13
Wales	7	100	0	16 273	105	0.65
Northern Ireland	5	100	0	6458	30	0.46

n, number of Trusts/Boards/Social Care Trusts in the region.

Inter-country pairwise comparisons using χ^2^ tests of significance (p<0.008) found Scotland to have a significantly higher hospital autopsy rate than each of the other countries (p<0.0001). Other pairwise comparisons failed to achieve significance (table 2).

**Table 2:** Summary of inter-country pairwise χ^2^ statistical analysis

	p Value
England vs Scotland	<0.0001*
England vs Wales	0.018
England vs Northern Ireland	0.619
Scotland vs Wales	<0.0001*
Scotland vs Northern Ireland	<0.0001*
Wales vs Northern Ireland	0.105

*Statistically significant after Bonferroni correction (p<0.008).

Twenty eight samples were statistical high outliers, 20 from England, 6 from Scotland, 1 from Wales and 1 from Northern Ireland. The mean hospital autopsy rate is skewed by these outliers, which typically were large teaching hospitals or small specialist centres. The top 5% (n=7) of Trusts within England performed 47% of the country's autopsies and 75% of autopsies in Wales were performed in one health board.

Ninety-eight per cent of samples (n=164) had an autopsy rate of <5%, 86% (n=143) an autopsy rate <1% and 23% (n=38) of all samples did not perform a single autopsy in 2013 (figure 2B). This demonstrates that for a quarter of NHS Trusts/Boards in the UK, hospital autopsy is extinct and in only a fraction (1.8%) of specialist trusts do autopsy rates exceed 5%, the rate previously published for non-teaching hospitals.^1^ ^18^

Hospital autopsy rates in children's hospital NHS Trusts ranged from 0% to 21%. This higher figure is in agreement with other literature.^17^ ^18^

## Discussion

This study has demonstrated that the evident decline in hospital autopsy has continued, if not accelerated, over recent years and already the hospital autopsy is extinct in many NHS Trusts. With 23% of NHS Trusts/Boards having an autopsy rate of 0%, a large part of UK hospital autopsy is now performed in a small number of centres. These few demonstrate that if the provisions and attitudes allow, then hospital autopsy rates of the recent past are still achievable, despite recent legislative changes such as the Human Tissue Act 2004/2006. Trusts with higher autopsy rates tended to be small specialised centres or large teaching hospital Trusts; this influence was not measured in this study due to difficulties in defining a ‘teaching’ or ‘specialised’ Trust/Board. Given that 86% of Trusts/Boards in the UK now have a hospital autopsy rate of <1%, we must pose the question whether a revival in hospital autopsy is possible? In the near future, many of these organisations may join the 23% in which hospital autopsy is extinct, unless they implement those changes in policy and attitude present in the 1.8% of Trusts/Boards where hospital autopsy exceeds 5% of inpatient deaths?

The hospital autopsy rate in Scotland was significantly higher than the other countries (table 2). The causes of this are uncertain but may include variations in the Human Tissue Act and Authority in Scotland or a lower procurator fiscal (coronial) autopsy rate.

A number of Trusts/Boards gave some explanations as to why their autopsy rate was low, these commonly surrounded provision of facilities. For example, one Trust does not employ an onsite histopathologist or have its own autopsy facilities. However, some Trusts/Boards which themselves do not have onsite hospital autopsy facilities have an agreement with neighbouring Trusts/Boards to carry out their autopsies. From the results, there is evidence of remote island providers that continue to implement autopsy despite no local facilities but which transport cadavers via boat or aeroplane to a separate hospital for autopsy. Thus, a lack of facilities does not preclude hospital autopsy although may add significantly to the cost.

Future research should investigate the differences in Trust/Board policies, clinician attitudes, facilities, funding and local demographics to determine how significantly higher autopsy rates can be achieved.

The strength of this study lies in the nationwide approach to calculating contemporary hospital autopsy rates. Previous studies have focused on single hospitals or Trusts; given the demonstrated wide inter-Trust variation this approach may lead to significant errors. A weakness of this study was that some hospital trusts were unable to separate the data for deaths and autopsies for children and adults. Therefore, mean adult autopsy rates may be slightly over-reported, rates being generally higher among paediatric deaths.

In England and Wales, 94 455 coronial autopsies were performed in 2013^3^ yet only 1132 hospital autopsies were performed within the English and Welsh Trusts included in this study. Hospital autopsy now accounts for approximately 1.2% of total autopsies. With such low numbers, questions must be raised regarding the effect such decline has on quality assurance, public health, misdiagnosis (a key contributor to avoidable harm^19^ ^20^), audit and the teaching of both medical students and trainee pathologists. Hospital autopsy presents classic cases used to train junior pathologists, given that many coronial postmortems are not used for training. Training in hospital autopsy will become ever more important given the impending lack of pathologists to cover coronial autopsy. The aim of this paper is to raise awareness of the extent of the decline and to prompt discussion on its consequences. While debate continues over the value of hospital autopsy in medical practice, if action is not taken imminently, the practice may disappear.

**Take home messages**

- The decline in hospital autopsies has continued throughout the UK in recent years.
- The mean hospital autopsy rate in 2013 in the UK was 0.69% of hospital deaths.
- Hospital autopsy is extinct in 23% of all UK NHS Trusts and is endangered in the remaining.
- Further research into the impact on patient safety, audit, research, public health and teaching is required.
